# Supplementary material for: RNA sequencing reveals the emerging role of bronchoalveolar lavage fluid exosome lncRNAs in acute lung injury
Source: PeerJ. 2022 Mar 30;10:e13159. doi: 10.7717/peerj.13159 (PMC8976476; doi:10.7717/peerj.13159)
Supplement: Supplemental Information 2 [file peerj-10-13159-s002.zip › Raw Data 20211020/High-throughput sequencing data upload information.docx]

1. High-throughput sequencing data upload link address: <https://www.ncbi.nlm.nih.gov/guide/sequence-analysis/>
2. Accession number:

Control Group: NB1:SRR11892823

NB2:SRR11892822

NB3:SRR11892821

NB5:SRR11892820

Test Group: SB1:SRR11892819

SB2: SRR11892826

SB3:SRR11892825

SB5:SRR11892824
