# Supplementary figures and images for: RNA sequencing reveals the emerging role of bronchoalveolar lavage fluid exosome lncRNAs in acute lung injury
Source: PeerJ. 2022 Mar 30;10:e13159. doi: 10.7717/peerj.13159 (PMC8976476; doi:10.7717/peerj.13159)

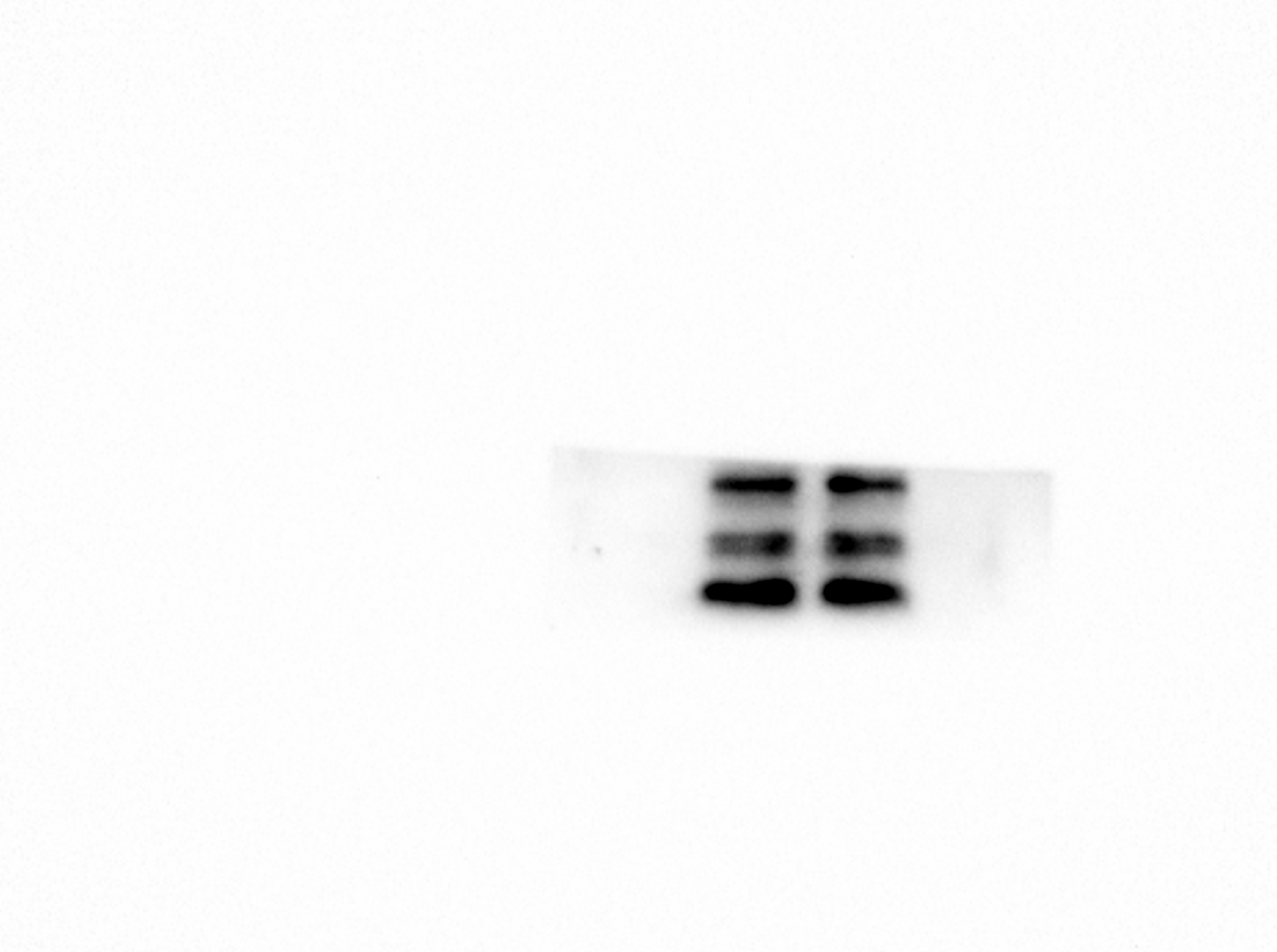

Supplement: Supplemental Information 2 [file peerj-10-13159-s002.zip › Raw Data 20211020/Figure 3/2-CD63 10s.tif]

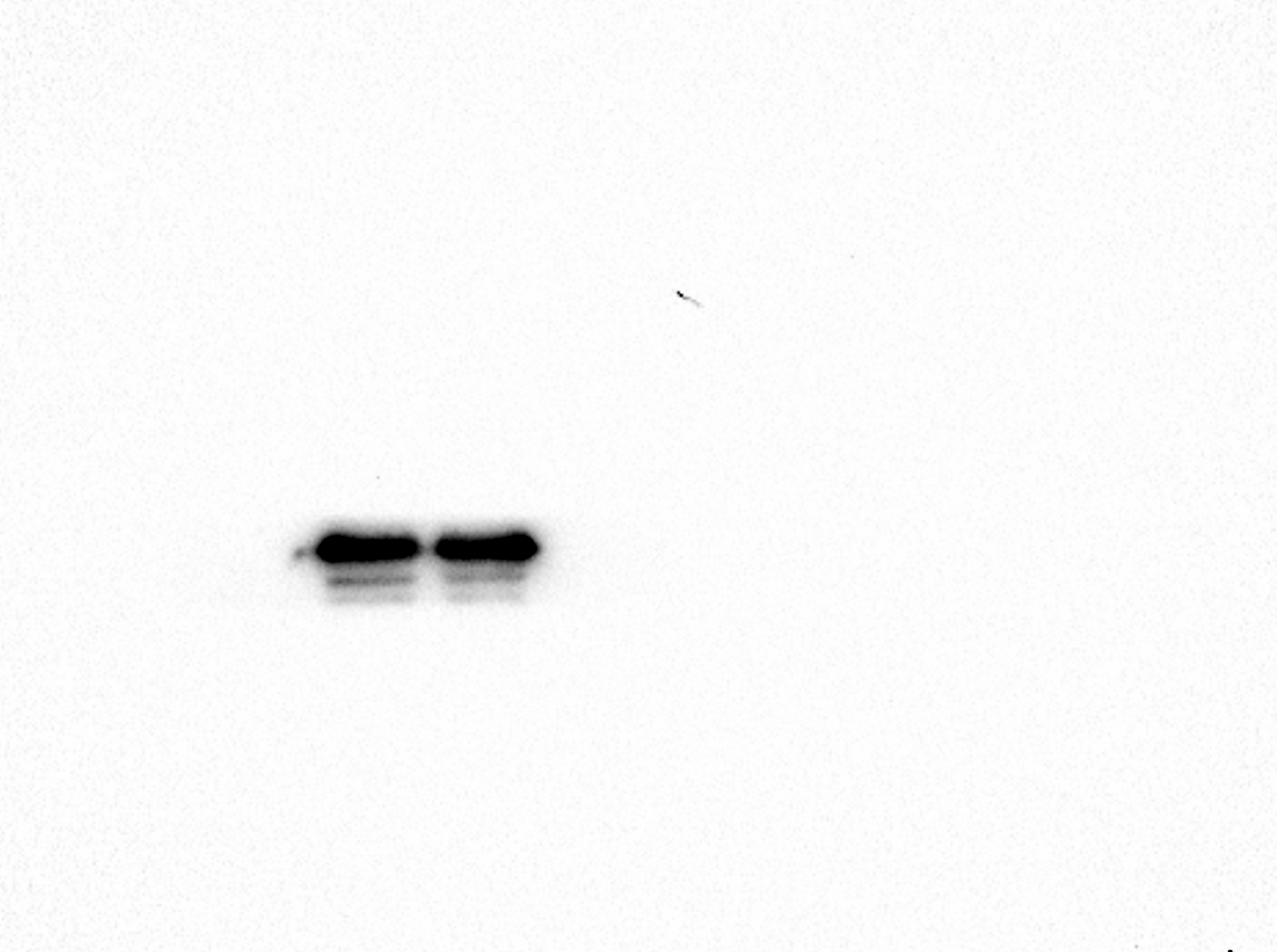

Supplement: Supplemental Information 2 [file peerj-10-13159-s002.zip › Raw Data 20211020/Figure 3/2-GA 10s.tif]

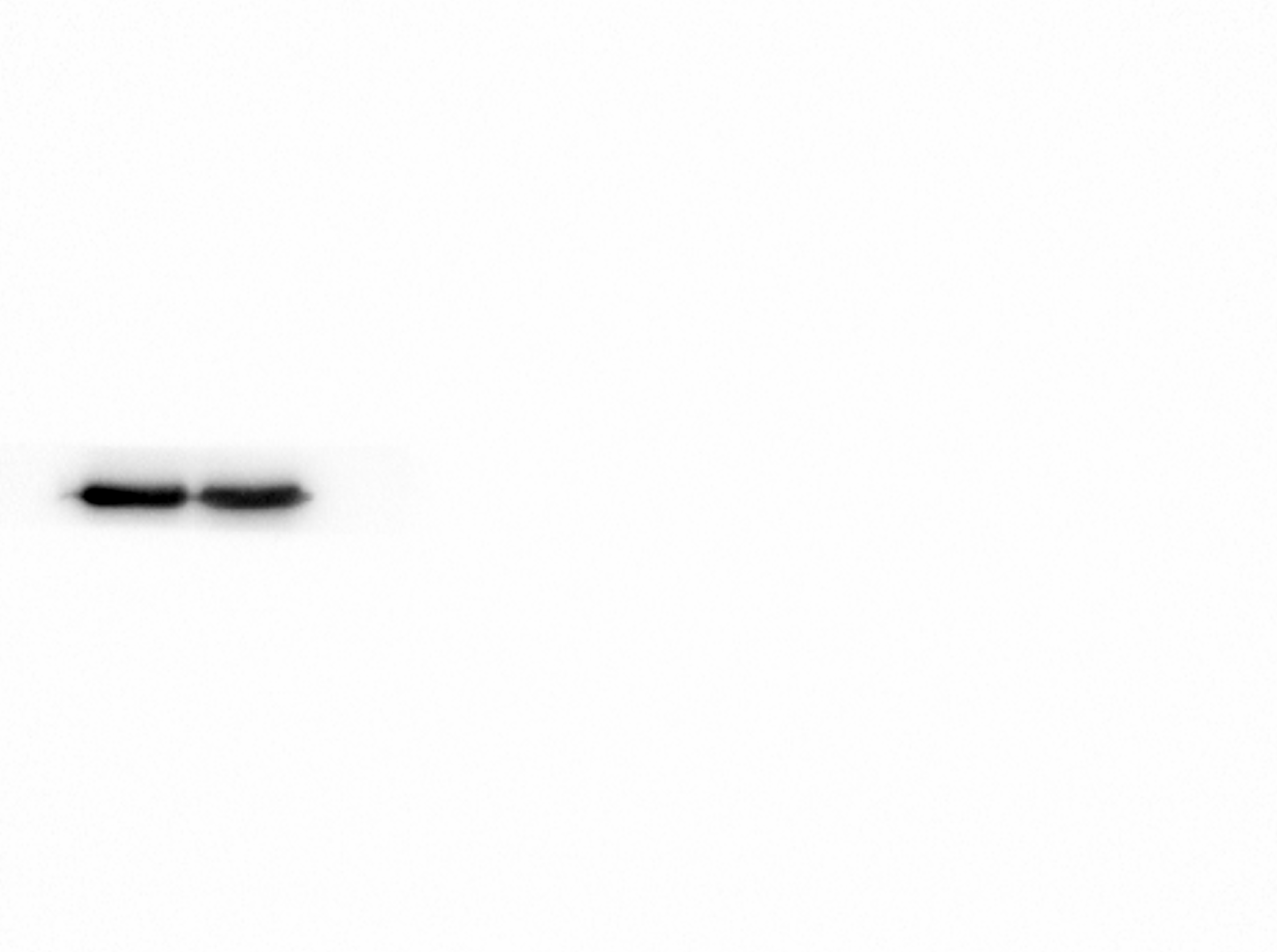

Supplement: Supplemental Information 2 [file peerj-10-13159-s002.zip › Raw Data 20211020/Figure 3/2-Tsg101 0.5s.tif]

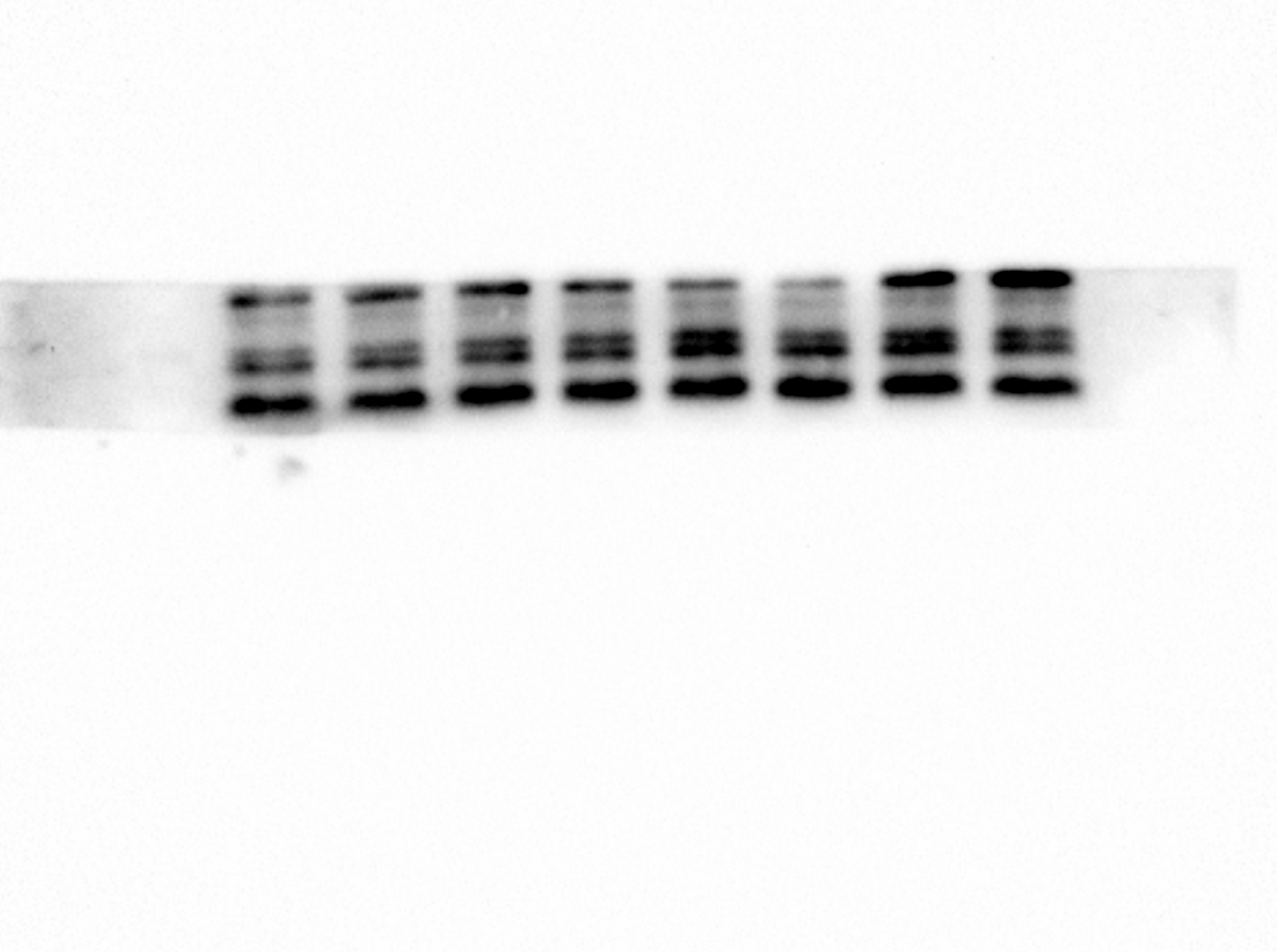

Supplement: Supplemental Information 2 [file peerj-10-13159-s002.zip › Raw Data 20211020/Figure 3/CD63 20s.tif]

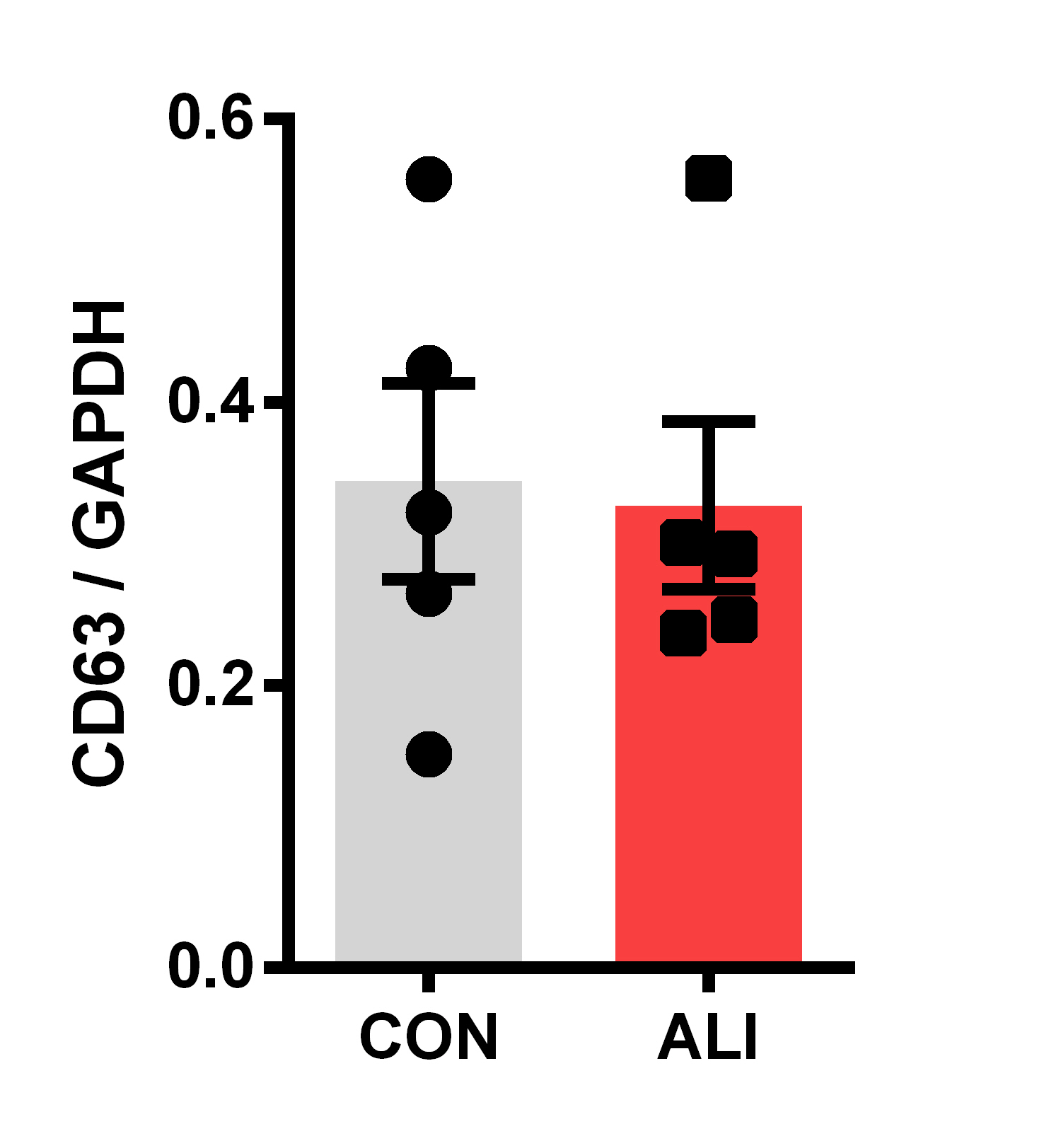

Supplement: Supplemental Information 2 [file peerj-10-13159-s002.zip › Raw Data 20211020/Figure 3/CD63.jpg]

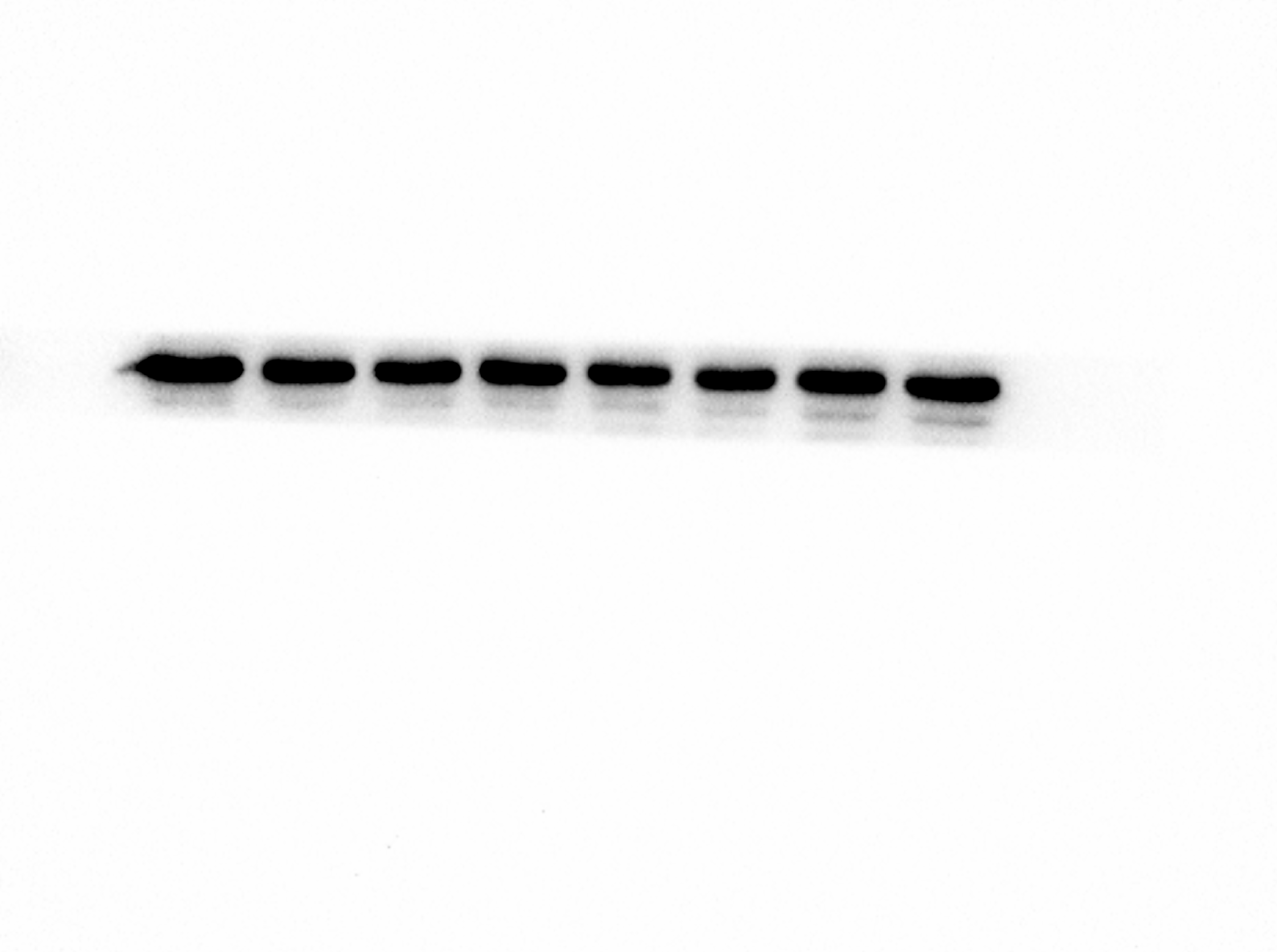

Supplement: Supplemental Information 2 [file peerj-10-13159-s002.zip › Raw Data 20211020/Figure 3/GA 6s 2.tif]

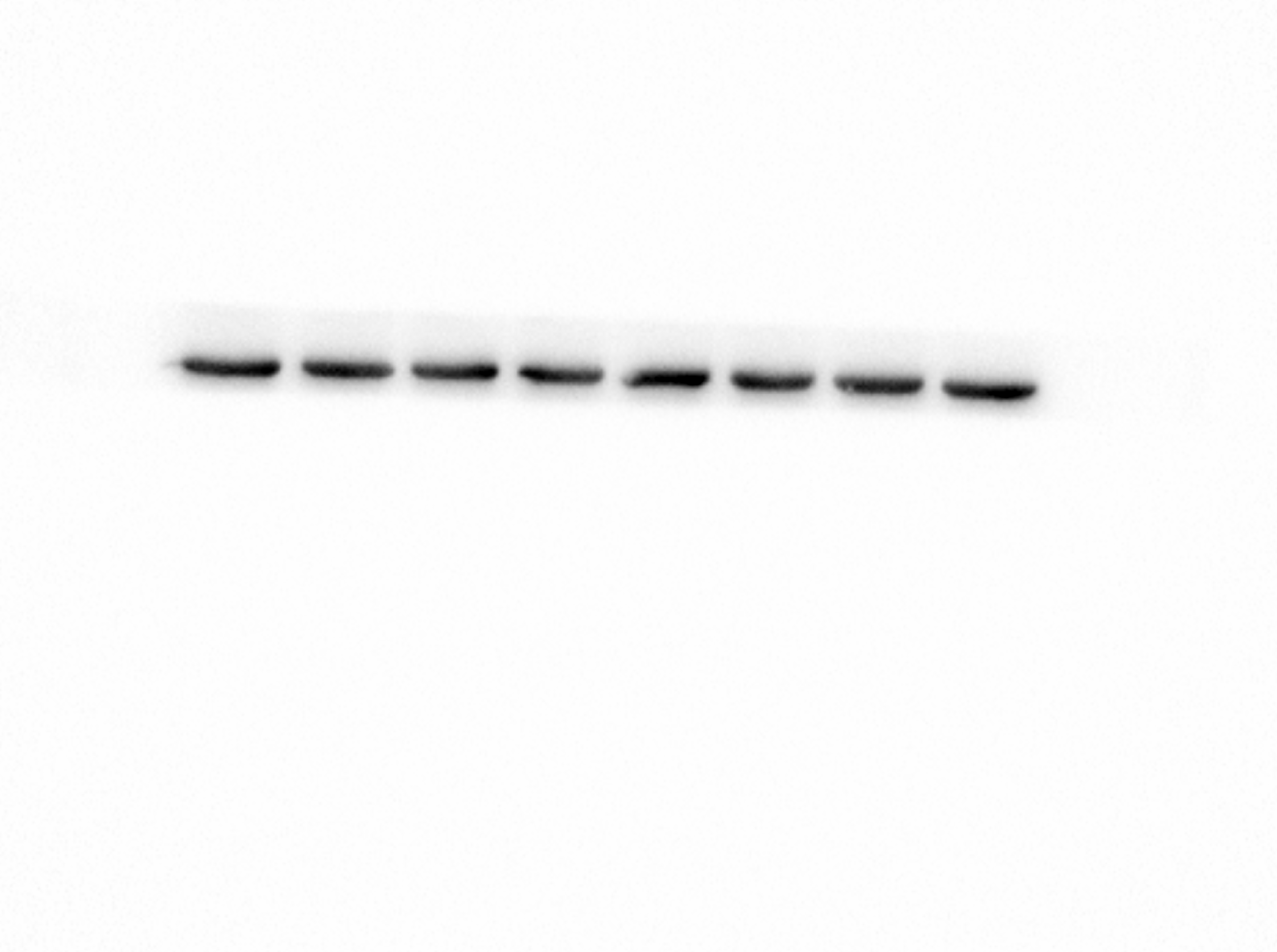

Supplement: Supplemental Information 2 [file peerj-10-13159-s002.zip › Raw Data 20211020/Figure 3/Tsg101 0.5s 2.tif]

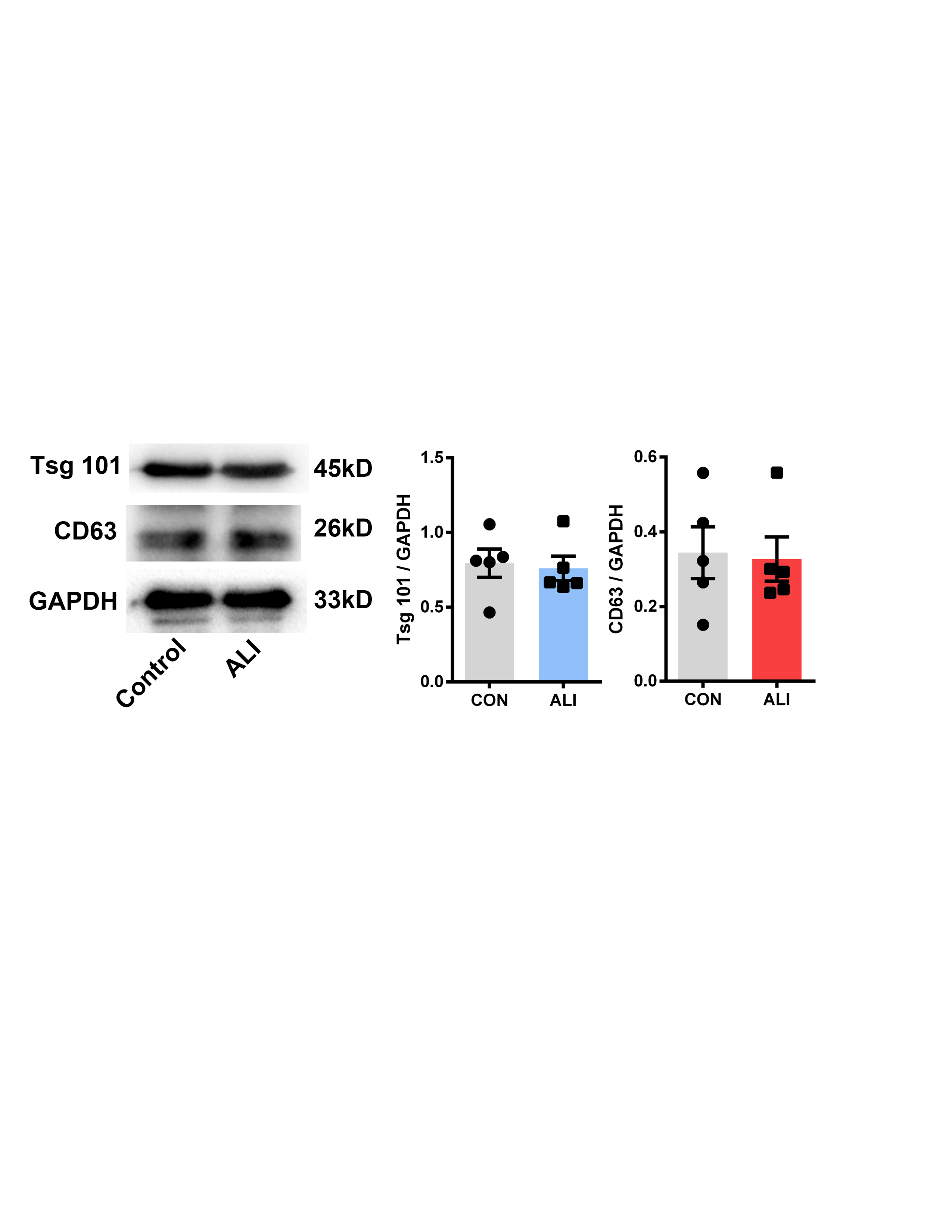

Supplement: Supplemental Information 2 [file peerj-10-13159-s002.zip › Raw Data 20211020/Figure 3/Tsg101 CD63.tif]

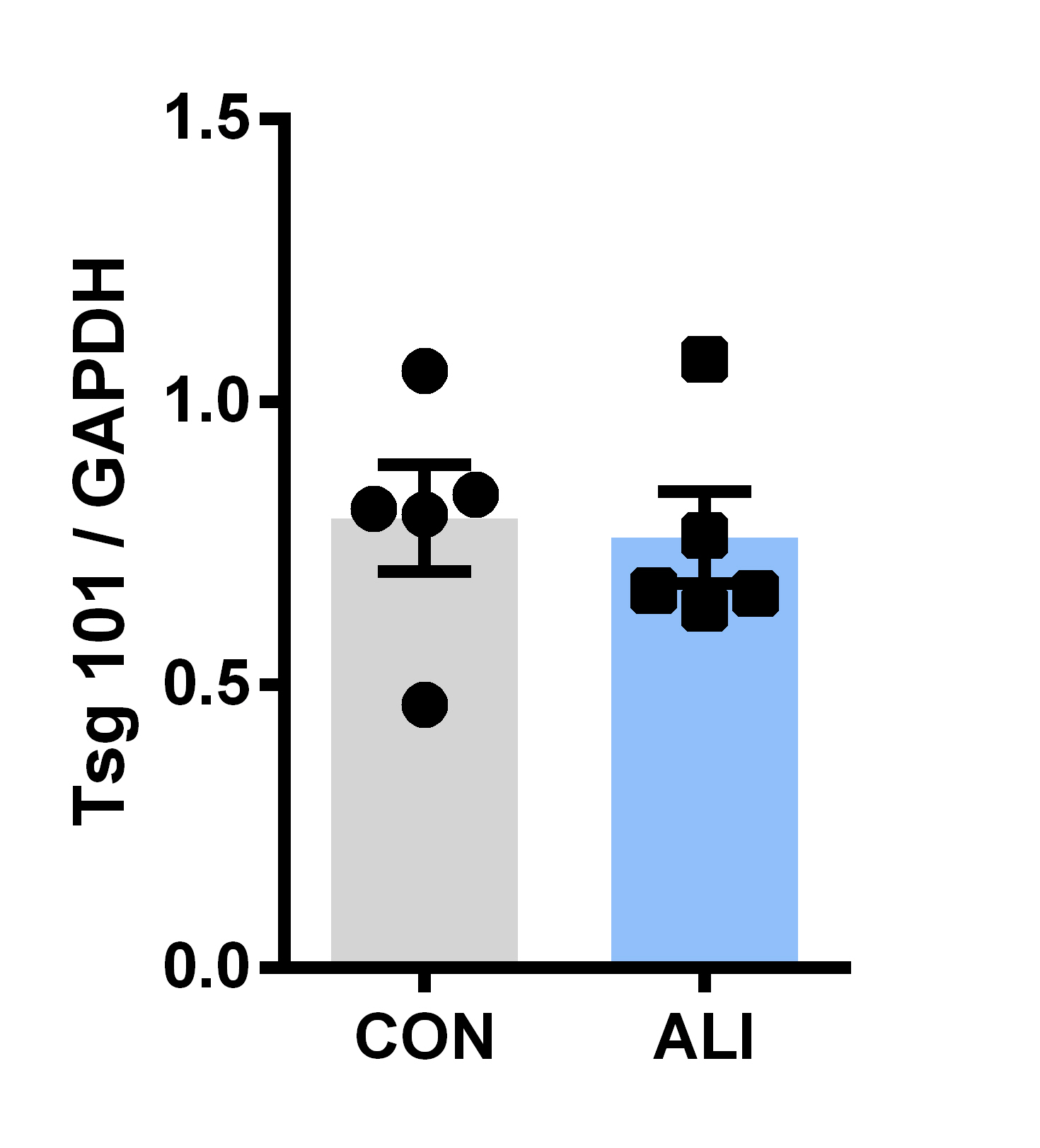

Supplement: Supplemental Information 2 [file peerj-10-13159-s002.zip › Raw Data 20211020/Figure 3/Tsg101.jpg]

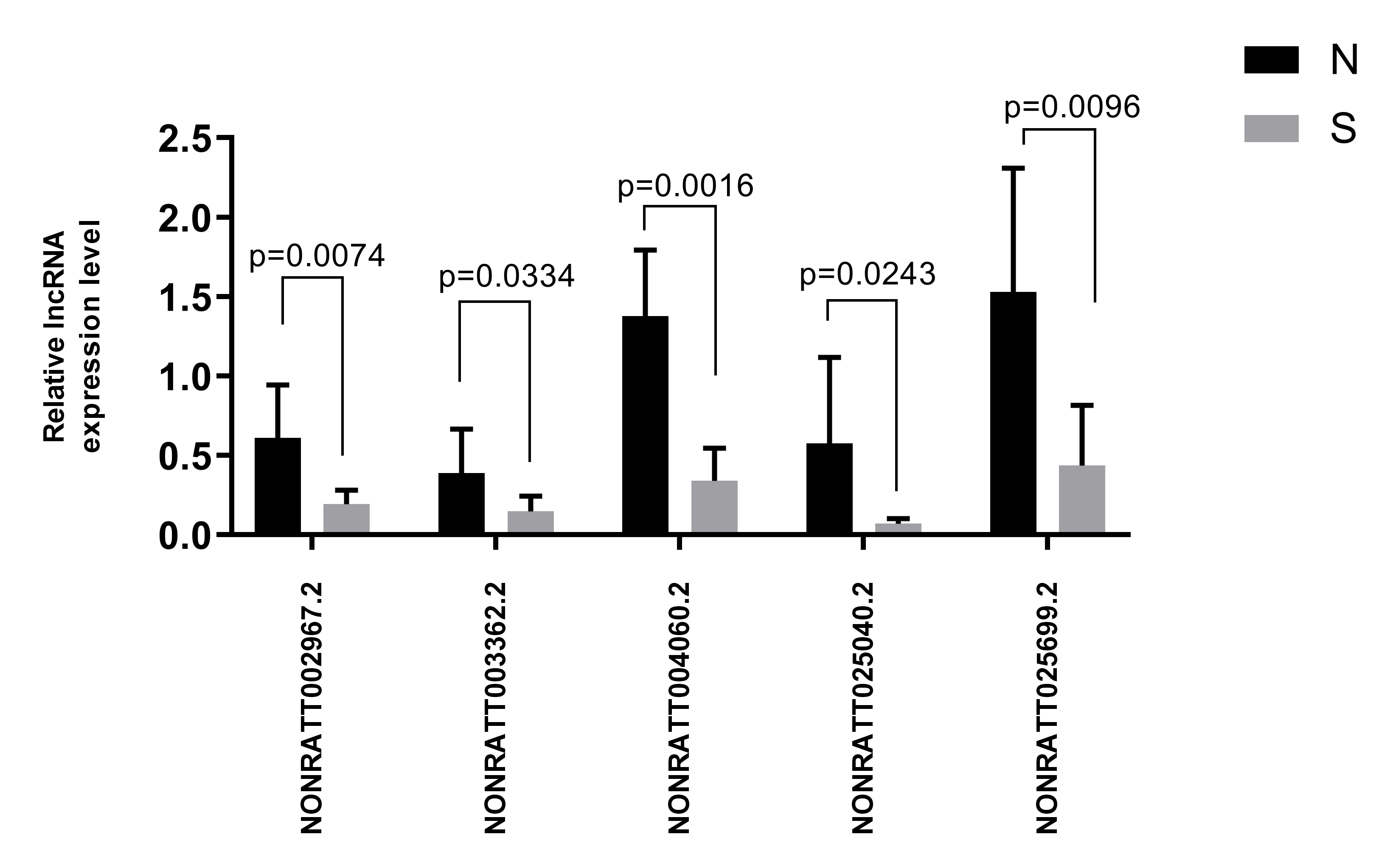

Supplement: Supplemental Information 2 [file peerj-10-13159-s002.zip › Raw Data 20211020/Figure 6/Figure 6.tif]
